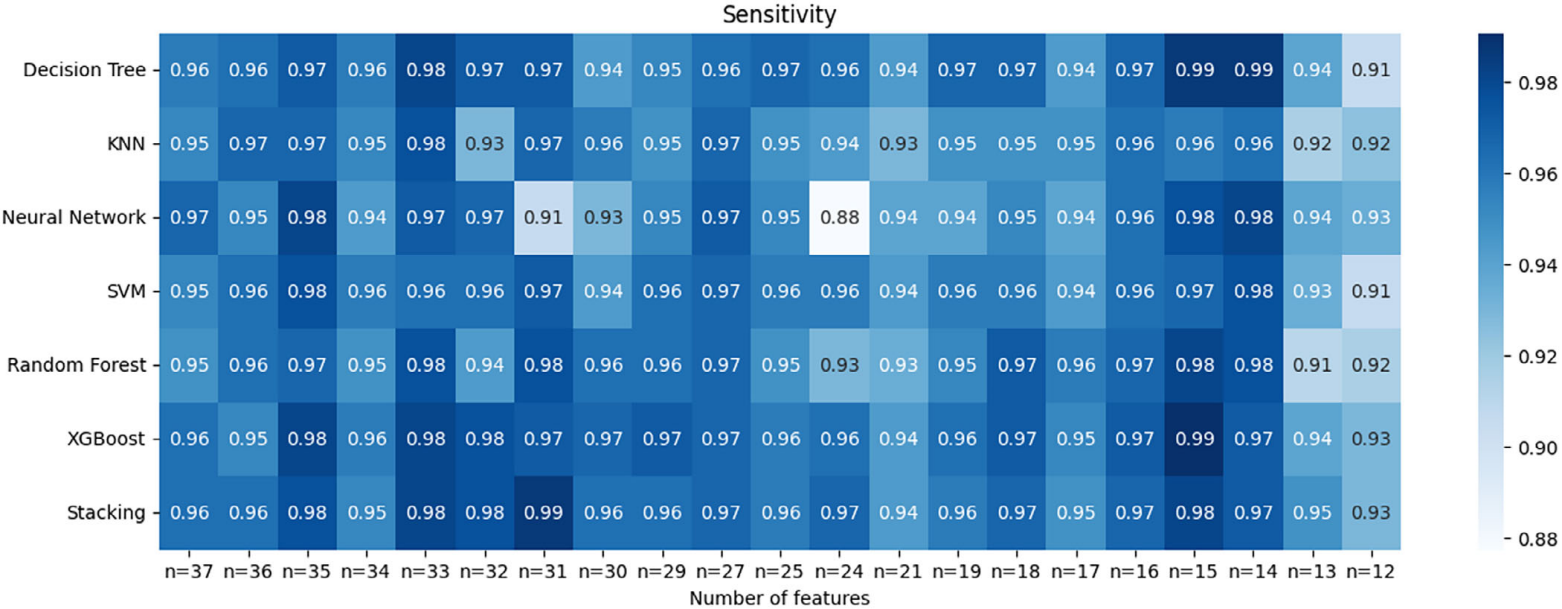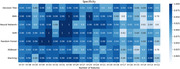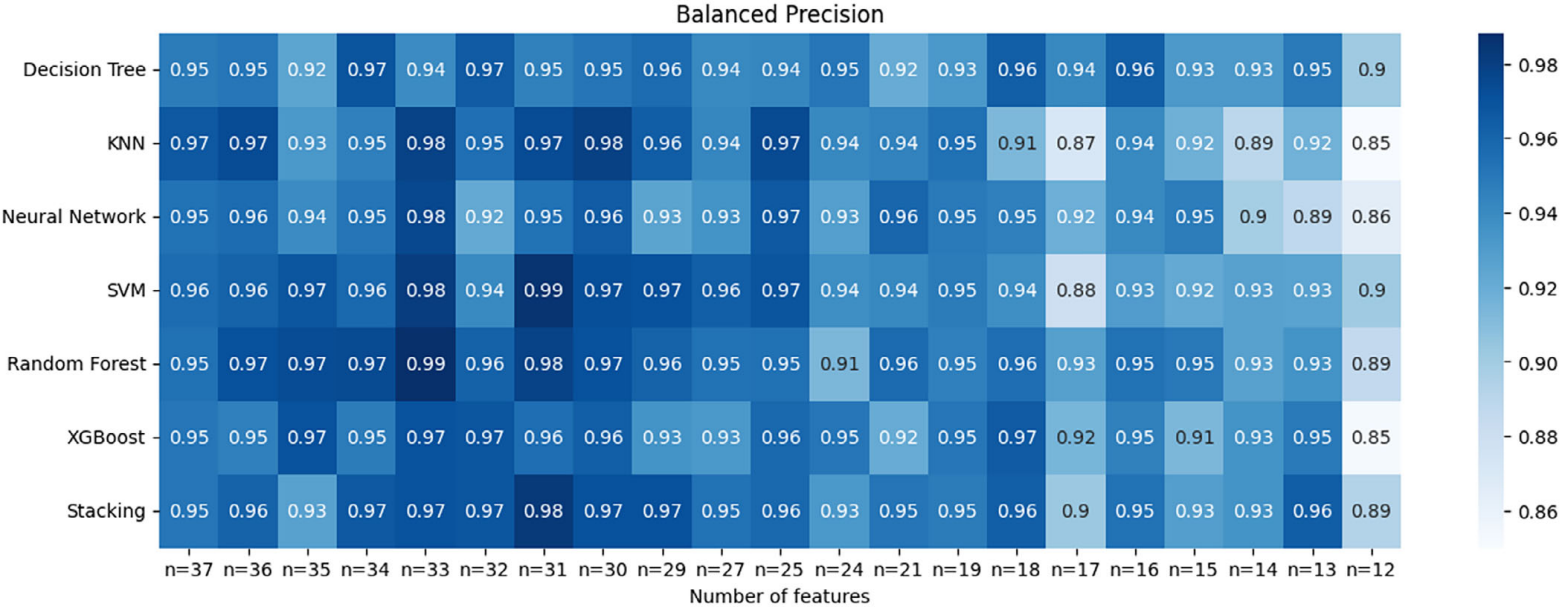# Improving diagnostic accuracy: development of an artificial intelligence model to aid in the diagnosis of Alzheimer’s disease dementia

**DOI:** 10.1002/alz.090115

**Published:** 2025-01-09

**Authors:** Valeria Guerra Espinosa, Juan Carlos Arbelaez Maestre, Daniela Zabaleta, Mateo Ruiz Espinosa, Francisco Lopera, David Fernando Aguillon, Sofia Guerra Espinosa

**Affiliations:** ^1^ Infovital, Envigado Colombia; ^2^ Universidad de Antioquia, Medellin Colombia; ^3^ Grupo de Neurociencias de Antioquia, Facultad de Medicina, Universidad de Antioquia, Medellín Colombia; ^4^ Grupo de Neurociencias de Antioquia, Universidad de Antioquia, Medellín Colombia

## Abstract

**Background:**

Currently, the diagnosis of Alzheimer’s disease dementia (ADD) is determined based on clinical criteria, as well as specific imaging and cerebrospinal fluid (CSF) biomarker profiles. However, healthcare professionals face a variety of challenges that hinder their application, such as the interpretation and integration or large amounts of data derived from neuropsychological assessment, the importance attributed to each source of information and the impact of unknown variables, among others. Therefore, this research focuses on the development of a computerized diagnostic tool based on Artificial Intelligence (AI), to strengthen the capacity of healthcare professionals in the identification and diagnosis of ADD.

**Method:**

During this research, all phases of the Cross‐Industry Standard Process for Data Mining (CRISP‐DM) methodology were applied. Specifically, seven types of machine learning (ML) classifier algorithms were trained with data derived from the medical and neuropsychological evaluation of 870 people with and without ADD. Additionally, genetic algorithms (GAs) were used to choose the hyperparameters of six of the models and the best performing model was deployed through a graphical user interface.

**Result:**

A total of 147 ML models were trained to differentiate or classify between people with and without ADD. Using a random forest (RF) algorithm and 31 predictor variables we achieved a sensitivity of 98%, a specificity of 100% and an area under the curve (AUC) of 0,988.

**Conclusion:**

We trained and evaluated a variety of ML models, highlighting the accuracy of a RF algorithm together with the use of GAs, to distinguish people with and without ADD. Additionally, we deployed the model with the best performance metrics through a graphical user interface in order to promote its future use in clinical settings.

Although these results are promising, the ongoing need to evaluate and re‐train the model, as well as the ethical considerations of its implementation, are well acknowledged.